# The Impact of Sex on the Outcomes of Prosthetic Joint Infection Treatment with Debridement, Antibiotics and Implant Retention: A Systematic Review and Individual Patient Data Meta-analysis

**DOI:** 10.5435/JAAOSGlobal-D-22-00102

**Published:** 2022-11-09

**Authors:** Annabelle L. Choong, Cade Shadbolt, Emma Choong, Tim Spelman, Ernesto Muñoz-Mahamud, Jaime Lora-Tamayo, Katy Kim, Marjan Wouthuyzen-Bakker, Mark Spangehl, Methee Chayakulkeeree, Simon W. Young, Peter F. M. Choong, Michelle M. Dowsey

**Affiliations:** From the Department of Surgery, The University of Melbourne, Melbourne, Australia (A.L. Choong, Shadbolt, E. Choong, Spelman, P.F.M. Choong, Dowsey); Department of Orthopaedics and Trauma Surgery, Bone and Joint Infection Unit, Hospital Clinic Barcelona, University of Barcelona, Barcelona, Spain (Muñoz-Mahamud); Department of Internal Medicine, Hospital Universitario, Madrid, Spain (Lora-Tamayo); Department of Medicine, The University of Auckland, Auckland, New Zealand (Kim); Department of Medical Microbiology and Infection Prevention, University Medical Center Groningen, University of Groningen, Groningen, the Netherlands (Wouthuyzen-Bakker); Department of Orthopaedic Surgery, Mayo Clinic Arizona Phoenix, AZ (Spangehl); Department of Medicine, Faculty of Medicine Siriraj Hospital, Mahidol University, Bangkok, Thailand (Chayakulkeeree); Department of Orthopaedic Surgery, University of Auckland, Auckland, New Zealand (Young); and Department of Orthopaedics, St. Vincent’s Hospital, Melbourne, Australia (P.F.M. Choong, Dowsey).

## Abstract

The influence of sex on the failure of débridement antibiotics and implant retention (DAIR) for treating prosthetic joint infection (PJI) is important for decision-making, patient counseling, and equitable health care. However, very few studies in the orthopaedic literature conduct sex-specific analyses.

**Aim:**

The primary aim was to determine whether sex influences treatment success after DAIR.

**Methods:**

A systematic review and individual patient data (IPD) meta-analysis was conducted. MEDLINE (Ovid), EMBASE (Ovid), Web of Science, and Google Scholar were searched, and IPD was requested via e-mail. Patients who underwent DAIR after developing PJI within 12 months of a primary total hip or knee arthroplasty were included in the analysis. Treatment failure was defined by the Delphi International Consensus criteria. Adjusted odds ratios for treatment failure were calculated using a mixed-effects logistic regression.

**Results:**

The study collected and analyzed IPD of 1,116 patients from 21 cohorts. The odds of treatment failure were 29% lower in women (odds ratio, 0.71; 95% CI 0.54 to 0.017; *P* = 0.017), after adjusting for duration of symptoms >7 days and *Staphylococcus aureus* infection (methicillin-susceptible *Staphylococcus aureus* or any infection with *S aureus*). None of the 64 studies included in the systematic review conducted a sex-specific analysis.

**Conclusion:**

For patients who developed a PJI within 1 year postsurgery, females have lower odds of DAIR failure than males. Other factors also have varying effects on outcome for men and women. It is essential to implement sex-specific analysis in orthopaedic research.

Prosthetic joint infection (PJI) remains one of the most devastating complications of total joint arthroplasty. The morbidity and economic burden associated with PJI is profound, approximating almost US$100,000 for inpatient costs and an additional US$50,000 for outpatient costs per infection.^1^

The revision implantation of a new prosthesis (one-/two-stage revision) after infection is widely used and well established. However, this technique is invasive and associated with significant morbidity, immobilization, and prolonged hospitalization.^2^ In addition, the cost of treating PJI using a two-stage revision is approximately 6-fold the cost of the primary implantation.^1^ This contrasts markedly with débridement antibiotics and implant retention (DAIR) where patients undergo less invasive surgical interventions, potentially reducing costs and risk of procedure-related morbidity. The usage of DAIR to treat PJI remains controversial with argument whether a failed DAIR increases the risk of an unsuccessful subsequent two-stage arthroplasty, resulting in loss of bone stock, economic burden and future uncontrolled infection or not.^3,4^

How sex differences affect certain diseases, injuries, and response to treatment is also important. For example, women have a higher prevalence of osteoarthritis^5^; female athletes have a significantly higher risk for anterior cruciate ligament injury compared with males,^6^ and significant differences have been noted for orthopaedic surgery outcomes when comparing differences in patient sex.^7^ In 2013, experts at the International Consensus Meeting on PJIs agreed that male sex significantly increased the risk of developing PJI after TJA, compared with female sex,^8^ a finding also supported by large international registry-based research in which the risk has been estimated as high as 2.4 times greater for males (95% CI 1.8 to 3.1 *P* < 0.001).^9^

The use of DAIR is becoming a popular treatment for early PJI and a target practice across the globe.^10^ Although the effectiveness of DAIR has been explored in large registry studies using aggregated data,^11^ it is noted that the common method of aggregating male and female data has been shown to mask existing responses to treatment. This might be due to unaccounted data, such as sex-associated covariates, including age, comorbidities, and obesity.^12^ Consequently, this could potentially confound information related to the safety and efficacy of treatment. In addition, sample size limitations in cohort studies have impaired the ability to draw any significant conclusion relating to sex differences.^13^ Identifying potential sex differences for DAIR success rates may have important implications for treatment decisions, patient-doctor communication, patient expectation, and economic modeling. This also answers a call to action raised by a recent study that retrospectively reviewed abstracts listed in the American Academy of Orthopaedic Surgeons annual meeting programs from 2006 to 2013 and found that only 5.4% of the research presented reported results of a sex-specific analysis.^14^ Moreover, in accordance with the US Food and Drug Administration's guidelines for sex-specific studies,^12^ an individual patient data (IPD) meta-analysis is necessary for sufficient enrollment of both sexes, disaggregation of data by sex, and subsequent exploration of sex-associated covariates. Retrieving IPD and seeking additional information from the investigators responsible for the original studies reduces the risk of the bias associated with aggregated data reviews.^15^ As such, we conducted a systematic review and IPD meta-analysis to answer the question of whether a sex-specific difference existed between males and females after DAIR for acute PJIs.

## Methods

The systematic review and IPD meta-analysis was conducted using a predefined protocol registered in the PROSPERO international prospective register of systematic reviews (CRD42020201135) and in accordance with the recommendations by the IPD Meta-analysis Methods Group of the Cochrane Collaboration^16^ and the Preferred Reporting Items for Systematic Reviews and Meta-Analyses of Individual Participants Data (PRISMA-IPD) guidelines^17^(Supplemental Appendix 1, http://links.lww.com/JG9/A246).

## Individual Patient Data Collection

We sought published IPD from studies identified through a transparent, systematic search. The MEDLINE (Ovid), EMBASE (Ovid), and Web of Science databases were searched from their inception to July 2020. A narrower set of supplementary searches were included using Google Scholar because this source frequently captures eligible studies that are missed by other databases. The first 500 articles for (i) hip and (ii) knee were exported using Harzing Publish or Perish. Keywords were tailored to each database that were used in the search. Using Medical Subject Heading, the following conceptual groups were included in the search strategy: “Prosthesis-Related Infections” OR “Wound Infection” OR “Sepsis” OR “Surgical Wound Infection” AND “Debridement” AND “Arthroplasty, Replacement” OR “Arthroplasty” OR “Arthroplasty, Replacement, Hip” OR “Arthroplasty, Replacement, knee”. Articles that referenced (forward citation tracking) or were referenced (backward citation tracking) by included studies and relevant published literature reviews were searched to identify additional eligible studies. Further details on the search strategy are presented in Supplemental Appendix 2 (http://links.lww.com/JG9/A246).

In addition, investigators of eligible studies with aggregated data were contacted via e-mail and invited to share anonymized IPD that would be combined with similar data from other contributing studies. The e-mail included a detailed summary of the study, an attachment of the PROSPERO protocol (CRD42020201135), clear instructions on which data were needed (Supplemental Appendix 5, http://links.lww.com/JG9/A246), the definition of each item, and a standardized Microsoft Excel data spreadsheet. A subsequent follow-up e-mail was sent 3 weeks after the initial contact. If the authors did not respond by the set time point or refuse to contribute IPD, these studies remained in the systematic review but were excluded from the meta-analysis.

## Eligibility Criteria

Studies were included if they met the following criteria (Table 1).

**Table 1:** Eligibility Criteria

Inclusion	Exclusion
• Consisted of a cohort of ≥5 patients • Generally unselected patients who developed PJI of the hip or knee within 12 months of the index TJA • Defined PJI in accordance with (1) MSIS,^18^ IDSA,^19^ CDC,^20^ or ICM criteria^8^ (Supplemental Appendix 3, http://links.lww.com/JG9/A246), (2) cited by other published criteria, or (3) provided their own definition, which aligned with previously published validated definitions • Reported treatment outcome after DAIR (success or fail) • Mention of treatment outcome for male and female participants.	• They had less than 12 mo of follow-up, unless failed treatment before the time cutoff • The index surgery was exclusively nonelective TJA (eg, hip fracture), revision arthroplasty, hemiarthroplasty, unicompartmental/partial arthroplasty, or mega-/endoprosthesis • They had undergone procedures between the initial TJA and DAIR procedure • Only studies published from 2000 onward were included as it was unlikely that attempts to contact authors of publications greater than 20 years old yield IPD

CDC = Centers for Disease Control and Prevention, ICM = International Consensus Meeting, IDSA = Infectious Diseases Society America, IPD = individual patient data, MSIS = Musculoskeletal Infection Society

### Selection Process

**Figure 1:**
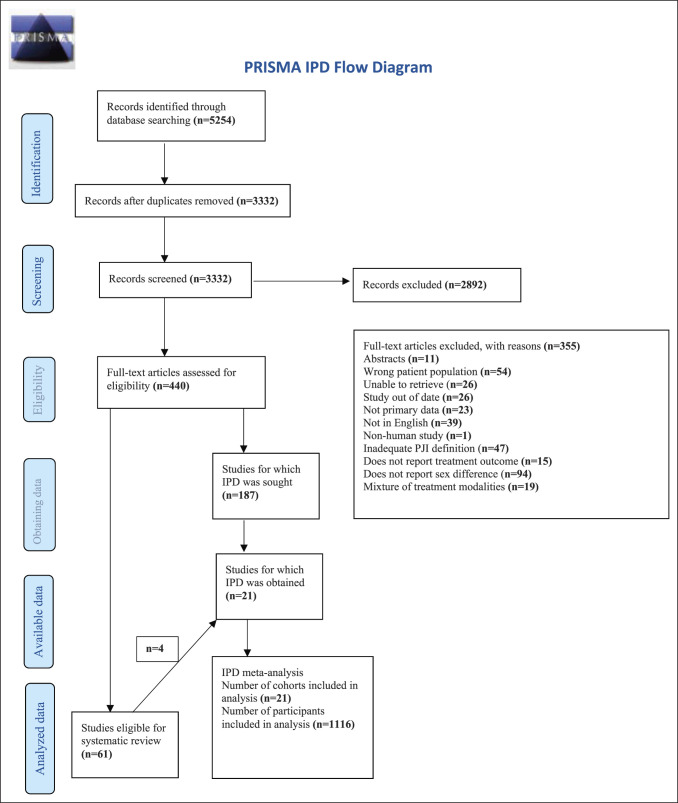
Preferred Reporting Items for Systematic Reviews and Individual Patient Data Meta-Analyses flow diagram of the search algorithm.

One reviewer (A.C.) screened, selected studies, assessed the quality of the studies, extracted data, and synthesized the results. Two additional reviewers (E.C. and C.S.) assisted with the screening and study selection process. Disagreement was resolved through discussion between these reviewers. A.C. obtained and managed shared data sets. When studies with an overlapping study cohort or database were identified, care was taken to determine whether the follow-up periods and characteristics of the separate reports were the same. If this was the case, it was ensured data in these studies were used only once for the analyses, but reference was made to all the publications in the PRISMA diagram (Figure 1). The study selection process was reported using a PRISMA-IPD flow diagram, with reasons for exclusion following full-text screening illustrated (Figure 1).

## Outcome Measure

The primary end point was a successful treatment outcome, which was defined according to the Delphi International Consensus criteria^21^: (1) infection eradication, characterized by a healed wound without fistula, drainage, or pain, no need for antibiotic suppression treatment, and no infection recurrence caused by the same or different organism(s) strain; (2) no subsequent unplanned surgical intervention for infection after DAIR; and (3) no occurrence of PJI-related mortality within 1-year follow-up. When outcome did not meet the above criteria, treatment was considered a failure.

### Data Extraction

The software tools, EndNote© and Covidence©, were used for deduplication, screening, and data extraction. A data template was developed in Microsoft Excel© 2016 (Microsoft). The following data were extracted: publication of paper, study design, patient characteristics, surgical characteristics, infection characteristics, outcome measures including duration of follow-up, and result summaries for sex differences (ie, odds ratio [OR] and 95% confidence intervals for each outcome measure and use of univariate/multivariate analysis). For studies that reported treatment outcome, only the results exclusive to DAIR were extracted.

### Risk of Bias

Methodological Index for Non-randomized Studies^22^ was used for nonrandomized surgical studies. The item “Unbiased evaluation of endpoints: blind evaluation of objective endpoints and double-blind evaluation of subjective endpoints” was omitted from the scoring system as it is not possible for a blind evaluation of treatment failure. As such, the remaining six items added up to a global ideal score of 14 for noncomparative studies. For the Joanna Briggs Institute checklist for case series,^23^ the item “Was statistical analysis appropriate?” was omitted from the checklist given that the data were reanalyzed and reported differently to fit the means of this study. Results of the critical appraisal process are presented in a summary table, which includes all articles in the systematic review and IPD meta-analysis.

## Individual Patient Data Integrity

IPD was checked for missing, invalid, out-of-range, and inconsistent items and for discrepancies with the original study. To reduce data availability bias, anomalies in the data set were queried and, where possible, resolved with the original authors to improve data quality. The Cochrane Handbook^16^ provided guidance in making an overall judgment about the risk of bias due to missing results. As such, multiple imputation was used for the missing data. Inevitably, the multiple studies included in an IPD meta-analysis collected and defined data in different ways. When authors were unwilling to prepare data in accordance with the prespecified format, the data were accepted and recoded as necessary, which made it possible to harmonize information, for example, definitions of acute PJI or treatment outcome, in a consistent manner to facilitate accurate pooling of IPD across studies. Studies that were within the eligibility criteria but IPD was unable to be obtained were excluded from the meta-analysis but remained included in the systematic review to explore the trends in sex-specific analysis and reporting in the joint infection literature.

### Ethics Approval

Ethics approval was granted by the St Vincent's Hospital (Melbourne) Human Research Ethics Committee (HREC-LRR 251/20).

### Statistical Analysis

Baseline categorical variables for women and men were summarized using frequency and percentage and compared between patient sex using a chi-square or a Fisher exact test. Baseline continuous variables were summarized using mean and SD or median and interquartile range and compared using a *t*-test or Wilcoxon rank-sum test as appropriate. The prevalence of treatment failure was estimated separately for women and men. A one-stage IPD meta-analysis hierarchical mixed-effects logistic regression was used to analyze the pooled data for associations between treatment failure and sex, adjusting for demographic, clinical, and treatment factors. An indicator variable representing the individual studies contributing IPD to the pooled analysis was modeled as a random effect. To examine the independent effects of sex on risk factor associations with treatment failure, variables found to be significantly associated with treatment failure on the unadjusted, univariable modeling were included as candidate explanatory variables in the adjusted, multivariable models. This modeling was analyzed and reported separately for women and men. For all analyses, *P* < 0.05 was considered significant. Because this was a sex-specific analysis, all IPD included in the analysis had at least the sex and treatment outcome available. Multiple imputation using chained equations was used to impute missing data for key explanatory variables using the MICE^24^ package in R. For variables included in the analysis, the proportion of missing data varied from 4 to 33%. If greater than 40% of data were missing, the variable was excluded from the analysis. Categorization for treatment failure at 3 and 6 months was used to conduct a sensitivity analysis on the primary analysis. All analyses were conducted in R version 3.6.3 (R Foundation for Statistical Computing) and Stata version 16.1 (StataCorp).

## Results

### Search Strategy

A total of 5,254 studies were identified through the search strategy. There were 3,332 remaining after the removal of duplicates. After title and abstract screening, 440 proceeded to full-text screening, and 355 were excluded from the review, with reasons stated. A total of 61 studies were included in the systematic review (Supplemental Appendix 4, http://links.lww.com/JG9/A246). IPD was sought from 21 studies consisting of 1116 patients which were included in the IPD-meta analysis, of 4 of these which were also included in the systematic review. The PRISMA flow diagram illustrates the results of the search (Figure 1).

### Risk of Bias and Individual Patient Data Integrity

The Methodological Index for Non-randomized Studies score for observational studies included in the systematic review and IPD meta-analysis ranged from 6 to 13 out of 14, Table S1 (Supplemental Appendix 7, http://links.lww.com/JG9/A246). No study comprised research with the explicit purpose to examine the influence of sex on the outcome of DAIR, with the consideration of sex differences in the title, hypothesis/research question, or Methods sections, therefore increasing data collection and measurement bias. As such, 0 studies prospectively calculated the study size to investigate differences between women and men, therefore increasing the risk of the effect of sex being disregarded. For 6/68 studies, it was unclear whether the study included consecutive patients, and three studies did not report this item. Notably, 5/68 studies had prospective collection of data; the remaining were retrospective large registry-based studies and therefore scored a 1 out of 2. Sixty-four of 68 studies clearly stated end points appropriate to the aim of the study. Forty-one of 68 studies adequately stated that loss to follow-up was <5%, 15 studies had >5% loss to follow-up, and 12 studies did not adequately address this. Although the some of the individual data within these 15 studies had a follow-up period of least 12 months or more following surgery, all 15 studies were still scored a 1 for ‘reported but inadequate’ given the primary outcome had inadequate follow-up.

Table S2 (Supplemental Appendix 7, http://links.lww.com/JG9/A246) shows the results of the JBI checklist for case series, in which 0 of 11 studies completely fulfilled the criteria. Seven studies scored 8/10 in which clinical information about the patients, that is, comorbidities, was unclear. In 1/11 study, the author referenced another study for the diagnostic criteria of PJI, and although that criteria aligned with our definition, the measured outcome for treatment success was not adequately addressed in the manuscript. In 2/11 studies, the consecutive and complete inclusion of participants was unclear. In 1/11 studies, the treatment outcome after DAIR was unclear because the authors only reported failed DAIR. No issues were identified in checking IPD

## Systematic Review

### Study Characteristics

The summary of baseline and clinical characteristics of the included studies is shown in Table 2. Fifty-seven studies were retrospective, and four were prospective. In aggregate, 6,588 DAIRs were performed between 1989 and 2017. Fifteen studies reported the outcomes of DAIR exclusively for knees, 6 studies for hips, 31 studies for knees and hips, and 10 included hips, knees, and other joints (eg, shoulders, ankles, and elbows). The mean or median age of patients at the time of DAIR was 42 to 87 years.

**Table 2:** Summary of Baseline Characteristics of Patients Undergoing DAIR

Characteristic	Number (%) of Patients			*P* Value
	Total N = 1,116	Males n = 532 (47.7%)	Females n = 584 (52.3%)	
Age, mean (SD), yr	68.4 (9.9)	66.3 (10.0)	70.2 (9.4)	**<0.001**
BMI (SD), kg/m^2^ (continuous)	30.0 (8.8)	29.3 (8.5)	30.6 (9.1)	**<0.001**
Obesity (BMI ≥ 30 kg/m^2^) (n, %) (categorical)	567 (50.8%)	257 (48.3%)	310 (53.1%)	0.111
Joint (n, %)				
Hip	460 (41.2%)	209 (39.3%)	325 (46.6%)	0.447
Knee	641 (57.4%)	316 (59.4%)	325 (55.7%)	
Treatment success (n, %)				
Overall	764 (68.5%)	344 (64.7%)	420 (71.9%)	**0.005**
Hips	318 (69.1%)	137 (65.6%)	181 (72.1%)	0.098
Knees	434 (67.7%)	202 (63.9%)	232 (71.4%)	**0.032**
Reason for failure (n, % of failures)				
Subsequent surgery	239 (68.7%)	124 (66.0%)	115 (71.9%)	0.671
Chronic antibiotic suppression	45 (12.9%)	27 (14.4%)	18 (11.3%)	
PJI-related mortality	3 (0.9%)	2 (1.1%)	1 (0.6%)	
Unknown/not recorded	61 (17.5%)	35 (18.6%)	26 (16.3%)	
Comorbidity (n, %)				
Hypertension	626 (56.1.0%)	273 (51.3%)	353 (60.5%)	**0.002**
CVD	214 (19.2%)	110 (20.7%)	104 (17.8%)	0.224
Diabetes	240 (21.5%)	124 (23.3%)	116 (19.9%)	0.162
COPD	121 (10.8%)	55 (10.3%)	66 (11.3%)	0.605
CRF	86 (7.7%)	50 (9.4%)	36 (6.2%)	**0.043**
Liver cirrhosis	31 (2.8%)	18 (3.4%)	13 (2.2%)	0.240
Malignancy	106 (9.5%)	45 (8.5%)	61 (10.5%)	0.258
RA	54 (4.8%)	18 (3.4%)	36 (6.2%)	**0.031**
Reason for index arthroplasty (n, %)				
Osteoarthritis	1,066 (95.5%)	508 (95.5%)	558 (95.6%)	0.028
Rheumatoid arthritis	16 (1.4%)	9 (1.7%)	7 (1.2%)	
Osteonecrosis	8 (0.7%)	7 (1.3%)	1 (0.2%)	
Degenerative joint disease	22 (2.0%)	7 (1.3%)	15 (2.6%)	
Other	4 (0.1%)	1 (0.2%)	3 (0.5%)	
Surgical characteristics				
Liner/mobile component exchange (n, %)	540 (48.4%)	273 (51.3%)	267 (45.7%)	0.062
Mean time from index surgery to infection (SD), d	57.4 (82.8)	67.0 (90.1)	48.6 (74.5)	**0.007**
Duration of symptoms—mean (SD), d	10.5 (37.1)	12.6 (50.5)	8.6 (17.3)	0.252
Parental antibiotic duration—mean (SD), d	24.6 (18.4)	24.4 (17.0)	24.7 (19.5)	0.925
Oral antibiotic duration—mean (SD), d	96.7 (151.6)	92.8 (141.0)	100.2 (160.6)	0.203
Total antibiotics duration—mean (SD), d	114.3 (150.6)	110.9 (140.5)	117.4 (159.2)	0.191
Microorganism (n, %)				
Gram-positive bacteria	900 (80.7%)	439 (82.5%)	461 (78.9%)	0.127
Gram-negative bacteria	177 (15.9%)	60 (11.3%)	117 (20.0%)	**<0.001**
Fungal	13 (1.2%)	3 (0.6%)	10 (1.7%)	0.074
Polymicrobial	300 (26.9%)	124 (23.3%)	176 (30.1%)	**0.010**
Culture negative	5 (0.5%)	1 (0.2%)	4 (0.7%)	0.215
*Staphylococcus*	681 (61.9%)	328 (62.5%)	353 (601.3%)	0.684
*S. aureus*	387 (34.7%)	193 (36.3%)	194 (33.2%)	0.299
MSSA	282 (25.3%)	143 (26.9%)	139 (23.8%)	0.222
MRSA	69 (6.2%)	25 (4.7%)	44 (7.5%)	0.050
*S epidermidis*	226 (20.3%)	110 (20.7%)	116 (19.9%)	0.769
MSSE	7 (0.6%)	3 (0.6%)	4 (0.7%)	0.714
MRSE	42 (3.8%)	23 (4.3%)	19 (3.3%)	0.733
*Streptococci spp*	289 (25.9%)	150 (28.2%)	139 (23.8%)	0.100
*Propionibacterium*	4 (0.4%)	2 (0.4%)	2 (0.3%)	1.000
*Enterococcus spp*	116 (10.4%)	43 (8.1%)	73 (12.5%)	**0.015**
*E. coli*	71 (6.4%)	29 (5.5%)	42 (7.2%)	0.228
*Enterobacter spp*	41 (3.7%)	18 (3.4%)	23 (3.9%)	0.614
*Proteus spp*	47 (4.2%)	11 (2.1%)	36 (6.2%)	**0.001**
*Pseudomonas spp*	34 (3.1%)	9 (1.7%)	25 (4.3%)	**0.015**
*Candida spp*	12 (1.1%)	3 (0.6%)	9 (1.5%)	0.112
Other	143 (12.8%)	48 (9.0%)	95 (16.3%)	<0.001
Follow-up (SD), months	25.7 (36.7)	29.9 (39.4)	21.9 (33.6)	<0.001

BMI = body mass index, COPD = chronic obstructive pulmonary disease, CRF = chronic renal failure, MSSA = methicillin-susceptible *Staphylococcus aureus*, MRSA = methicillin-resistant *Staphylococcus aureus*, RA = rheumatoid arthritis

### Sex and Débridement Antibiotics and Implant Retention

Three studies^25‐27^ reported male sex, and one study^28^ reported female sex as a significant predictor of failure. Two studies found that male sex was an independent predictor of failure, but differed as to whether the infection was early or late (male sex as a predictor for failure beyond 30 days after DAIR; adjusted HR, 1.85 95% CI, 1.18 to 2.91, *P* = 0.009 ^27^; male sex as an independent predictor of failure within 30 days after DAIR; adjusted OR 95% CI 2.48 1.19 to 5.19 *P* = 0.016).^26^ In contrast, Lowik et al^28^ observed 769 DAIRs after primary and revision TKA and THA and reported that female sex was an independent predictor for treatment failure (OR 1.89, 95% CI 1.14 to 3.13; *P* = 0.013). Notably, these studies were not exclusively investigating primary arthroplasties, and no study consisted of analysis, evaluation, and reporting of sex-specific data.

### Individual Patient Data Meta-analysis

#### Sex-specific Baseline and Follow-up Characteristics

The pooled sample consisted of 1,116 patients retrieved from 21 cohorts (Supplemental Appendix 6, http://links.lww.com/JG9/A246). Of these, summary baseline and follow-up characteristics of the patients with PJI of the hip and knee treated by DAIR that contributed to the analyses are shown in Table 2. Five hundred thirty-two patients (47.7%) were male, and 584 (52.3%) were female (Table 2). Overall, the failure rate of DAIR was 31.5%, with higher failure rates for men than women (35.3% versus 28.1%, *P* = 0.005). On average, female patients were older than male patients (70.2 versus 66.3 years, *P* < 0.001), had higher BMIs (30.6 versus 29.3 kg/m^2^, *P* < 0.001), hypertension (60.5% versus 51.3%, *P* = 0.002), and RA as a comorbidity (6.2% versus 3.4%, *P* = 0.031). Male patients had a longer mean time between index surgery to infection (67.0 versus 48.6 days, *P* = 0.007) and higher prevalence of chronic renal failure than females (9.4% versus 6.2%, *P* = 0.043). Sex-specific baseline and follow-up characteristics Line 297: “and polymicrobial infections (30.1% vs 23.3%, p=0.010).

#### Treatment Outcome

Women had lower crude rates of treatment failure than men (28.1% versus 35.3%, *P* = 0.005) (Table 2). In an unadjusted analysis, female sex was associated with a 32% reduction in the odds of treatment failure relative to male sex (OR, 0.68; 95% CI, 0.52 to 0.88; *P* = 0.004) (Table 3). Multivariate models were adjusted for *Staphylococcus epidermidis, Staphylococcus aureus,* methicillin-susceptible *Staphylococcus aureus* (MSSA), and duration of symptoms, based on their significance in the unadjusted analyses (Table 3). After adjusting for these variables, female sex was associated with a 29% reduction in the odds of treatment failure relative to male sex (OR, 0.71; 95% CI 0.54 to 0.94; *P* = 0.017).

**Table 3:** Unadjusted and Adjusted Odds Ratios for the Association of Sex and Risk of Treatment Failure

Variable	Total N = 1,116						Male n = 532 (47.6%)						Female n = 584 (52.4%)					
	Unadjusted			Adjusted			Unadjusted			Adjusted			Unadjusted			Adjusted		
	OR	95% CI	*P* Value	OR	95% CI	*P* Value	OR	95% CI	*P* Value	OR	95% CI	*P* Value	OR	95% CI	*P* Value	OR	95% CI	*P* Value
Sex (female)	0.68	0.52-0.88	**0.004**	0.71	0.54-0.94	**0.017**	N/A						N/A					
Joint (knee)	1.03	0.77-1.39	0.823				1.07	0.74-1.55	0.703				1.07	0.72-1.59	0.745			
Age, yr	0.99	0.98-1.01	0.238				0.99	0.98-1.01	0.558				1.00	0.98-1.02	0.774			
BMI, kg/m^2^ (continuous)	0.99	0.97-1.02	0.533				1.02	0.99-1.04	0.157				1.00	0.97-1.03	0.931			
Hypertension	0.77	0.59-1.01	0.056				0.84	0.59-1.19	0.321				0.84	0.57-1.24	0.373			
CVD	1.21	0.87-1.68	0.269				1.23	0.80-1.89	0.356				1.22	0.75-1.97	0.422			
COPD	1.11	0.73-1.68	0.623				1.15	0.65-2.04	0.641				1.11	0.62-1.99	0.718			
CRF	1.23	0.76-1.98	0.392				0.85	0.46-1.58	0.604				1.90	0.94-3.85	0.073			
Liver cirrhosis	1.07	0.49-2.32	0.864				0.51	0.17-1.58	0.244				2.32	0.76-7.12	0.142			
RA	1.66	0.94-2.94	0.083				0.70	0.24-1.98	0.497				3.11	1.55-6.25	**0.001**	3.25	1.61-6.55	**0.001**
Diabetes mellitus	1.29	0.94-1.75	0.110				1.10	0.73-1.68	0.640				1.51	0.97-2.36	0.069			
Time from index surgery to infection, d	1.00	1.00,10.00	0.143				1.00	1.00,1.00	0.467				1.00	1.00-1.00	0.418			
Duration of symptoms >7 d	1.56	1.19-2.05	**0.001**	1.49	1.13-1.98	**0.005**	1.42	0.99-2.03	0.056				2.02	1.39-2.92	**<0.000**	1.93	1.28-2.92	**0.002**
Mobile parts exchange (no)	1.09	0.83-1.44	0.525				1.02	0.71-1.45	0.924				1.31	0.89-1.93	0.176			
Gram-positive bacilli	1.23	0.84-1.79	0.292				1.35	0.82-2.21	0.243				0.88	0.52-1.47	0.614			
Gram-negative bacilli	0.88	0.60-1.29	0.505				0.90	0.51-1.60	0.727				1.01	0.63-1.64	0.958			
Fungal	18.48	2.08-164.4	**0.009**				Insufficient numbers						7.15	1.55-33.04	**0.012**			
Polymicrobial	0.94	0.69-1.27	0.682				0.96	0.63-1.47	0.855				0.88	0.58-1.34	0.552			
Culture negative	1.70	0.25-11.72	0.591				Insufficient numbers						1.50	0.12-18.21	0.749			
*Staphylococcus spp.*	1.25	0.90-1.72	0.180				1.28	0.88-1.85	0.201				0.94	0.61-1.45	0.792			
*S aureus*	1.81	1.35-2.41	**<0.000**	1.55	0.94-2.58	0.087	1.91	1.32-2.77	**0.001**	1.79	0.91-3.51	0.091	1.37	0.91-2.08	0.130			
MSSA	1.69	1.23-2.31	**0.001**	1.10	0.65-1.86	0.714	1.79	1.20-2.67	**0.004**	1.09	0.54-2.21	0.807	1.47	0.94-2.31	0.093			
MRSA	1.44	0.82-2.53	0.208				2.02	0.90-4.53	0.088				0.87	0.40-1.86	0.715			
*S epidermidis*	0.67	0.47-0.94	**0.022**	0.80	0.55-1.17	0.251	0.82	0.53-1.29	0.397				0.58	0.35-0.96	**0.035**	0.69	0.34-1.39	0.297
MSSE	3.19	0.67-15.26	0.147				Insufficient numbers						Insufficient numbers					
MRSE	0.88	0.42-1.83	0.734				1.26	0.53-3.01	0.606				0.67	0.19-2.32	0.530			
*Streptococci spp*	0.98	0.65-1.50	0.943				0.96	0.65-1.44	0.860				0.93	0.52-1.69	0.821			
*Propionibacterium*	0.73	0.07-7.53	0.795				1.84	0.11-29.61	0.667				Insufficient numbers					
*Enterococcus spp.*	1.01	0.66-1.54	0.970				1.00	0.52-1.93	0.999				1.08	0.62-1.88	0.784			
*E coli*	1.01	0.57-1.78	0.977				0.96	0.44-2.12	0.929				1.22	0.58-2.53	0.601			
*Enterobacter spp.*	0.76	0.36-1.60	0.463				0.92	0.34-2.48	0.863				0.58	0.19-1.77	0.337			
*Proteus spp.*	1.43	0.76-2.70	0.265				1.55	0.47-5.14	0.477				1.50	0.71-3.16	0.288			
*Pseudomonas spp.*	0.75	0.33-1.71	0.497				1.04	0.25-4.24	0.958				0.68	0.25-1.90	0.466			
*Candida spp.*	9.83	1.43-67.46	**0.020**	10.31	1.58-67.31	**0.015**	Insufficient numbers						5.96	1.33-26.74	**0.020**	4.89	1.03-23.31	**0.046**
Other	0.82	0.55-1.24	0.352				0.82	0.43-1.54	0.531				0.88	0.52,1.47	0.618			

^a^Only applies to a subgroup of patients, thus not included in the multivariate model. Bold denotes statistical significance.

BMI = body mass index, CI = confidence interval, COPD = chronic obstructive pulmonary disease, CRF = chronic renal failure, MSSA = methicillin-susceptible *Staphylococcus aureus*, MRSA = methicillin-resistant *Staphylococcus aureus*, OR = odds ratio, RA = rheumatoid arthritis

#### Factors Related to Treatment Outcome

##### Rheumatoid Arthritis

RA was not a significant indicator for treatment outcome in males. RA was a significant predictor of treatment failure for females in both the unadjusted (OR, 3.11; 95% CI, 1.55 to 6.25; *P* = 0.001) and adjusted (95% CI 1.61 to 6.55; *P* = 0.001) sex-stratified analysis. Females with RA were 3.25 times more likely to fail treatment than females without RA.

##### Duration of Symptoms

Duration of symptoms >7 days was not a significant indicator for treatment outcome in males. In an unadjusted analysis, duration of symptoms >7 days was a significant predictor for treatment failure for females (OR, 2.02; 95% CI, 1.39 to 2.92; *P* < 0.000). Adjusting for RA and *S epidermidis*, females with symptoms >7 days were 1.93 times more likely to fail treatment than females <7 days of symptoms.

#### Organisms

##### Staphylococcus epidermidis

Infections caused by *S epidermidis* were not a significant indicator for treatment outcome in males. In an unadjusted sex-stratified analysis, *S epidermidis* associated with a 42% reduction in the odds of treatment failure for females (95% CI, 0.35 to 0.96, *P* = 0.035), but was not significant in the adjusted model.

##### Staphylococcus aureus

In an unadjusted sex-stratified analysis, *S. aureus* was a significant predictor of treatment failure for males (OR 1.91; 95% 1.32, 2.77; *P* = 0.001), but did not remain significant in the adjusted analysis. Infections caused by *S aureus* were not a significant indicator for treatment outcome in females.

##### Methicillin-susceptible *Staphylococcus aureus*

In an unadjusted sex-stratified analysis, MSSA as a subgroup within *S aureus* infections was a significant predictor of treatment failure for males (OR, 1.79; 95% CI, 1.20 to 2.67; *P* = 0.004) but did not remain significant in the adjusted analysis. Infections caused by MSSA as a subgroup within *S aureus* infections were not a significant indicator for treatment outcome in females.

## Sensitivity Analysis

The sensitivity analysis was performed to examine sex differences in treatment outcomes for infections developed within 3 and 6 months, with largely similar results to the primary analysis (Supplemental Appendix 8, http://links.lww.com/JG9/A246).

## Discussion

This study used individual-level data from 1,116 patients to analyze sex differences in treatment outcomes after DAIR. Our results indicated that female sex was independently associated with a 29% reduction in the odds of treatment failure relative to male sex. This finding supports evidence from some^25–27^ but not all previous aggregated reviews.^28^ However, it is difficult to directly compare the current findings with previous work because this is the first IPD meta-analysis for this population. Mounting evidence has indicated that sex hormones influence immunity, suggesting that testosterone has a suppressive effect on the immune system, whereas estrogen has an enhancing effect,^29^ which might help explain the current observed sex difference. In addition, in our sample, female patients were on average older than their male counterparts (70.2 versus 66.3 years, *P* < 0.001), and older age has previously been associated with higher DAIR success rates.^30^

Importantly, this analysis revealed that the varying effect certain covariates have on treatment outcome differ between women and men. One example was the effect of RA as a comorbidity. Female patients with RA were 3.11 times more likely to have an unsuccessful DAIR compared with females without RA; however, this comorbidity was insignificant for failure in males. A previous Japanese study found that the use of biologic disease-modifying antirheumatoid drugs increased the risk for post-TJA acute infection compared with nonbiologic disease-modifying antirheumatoid drugs (OR, 5.69; 95% CI, 2.07 to 15.61, *P* = 0.0007).^31^ Perhaps the existing sex difference in the effect of RA and DAIR failure could be caused by a sex difference in the management and/or response to treatment for RA. Further research in this area is warranted. In some research, the most prominent difference in treatment outcome was observed using a symptom duration of seven days as the optimal cutoff time frame.^32^ When we dichotomized duration of symptoms, >7 days was a significant risk factor for failure for women but not men. Although dichotomizing the variable assists in simplifying the statistical analysis, there is a risk of valuable information being lost.

Body size is an established sex-associated covariate,^12^ and higher BMI has been associated as a major risk factor for PJI development^33^ and morbid obesity has been reported to increase the risk of DAIR failure.^34^ A recent DAIR study^35^ indicated that severely obese patients were more often of female sex than non–severely obese patients (74.2% versus 60.0%, *P* = 0.046). Similarly, our female cohort had slightly higher BMIs than their male counterparts (30.6 versus 29.3, *P* = 0.007). Surprisingly, however, BMI was not a significant indicator for treatment outcome within the aggregated or sex-stratified analysis. Although the type of microorganism was not a significant predictor for treatment failure, it must be noted that we were limited in these data as not all patients had the microorganism recorded. Further research is warranted.

## Strengths and Limitations

In accordance with established guidelines,^12^ this study collected, analyzed, and reported data separately for women and men. Supported by previous evidence and demonstrated by the results, aggregated data and sex-disaggregated data using IPD produce discrepant but more accurate results.^36^ Another major strength is the sample size that informed our IPD. This is one of the most extensive studies conducted using IPD for evaluating sex differences in treatment outcomes and the effect of variables of failure for males and females separately. The ability to conduct this type of sex-specific analysis in orthopaedic studies is typically difficult in single-institution studies. Continuing to strengthen international partnerships and encourage collaboration is essential to increase the amount of data needed to adequately investigate sex differences and to prosper and drive medical discovery. Additionally, the use of the Delphi International Consensus criteria21 enabled the definition of treatment success to be consistent across cohorts and reduce the likelihood of bias in the outcome of treatment success.

Despite the novelty and strengths of the current study, several limitations deserve consideration. One disadvantage of conducting an IPD meta-analysis is data availability and selection bias.^37^ Eighty-nine percent of the contacted authors (166 out of 187) did not respond or were unable to provide raw data. The risk of selection bias limits the generalizability of the results. Another limitation was that multiple studies failed to adequately report surgical characteristics, such as whether DAIR was performed with or without modular parts exchange or the number of débridements that were performed as part of the treatment protocol. Although unreported preferences may be for at least liner exchanges, the study by Lowik et al, which accounted for a substantial number of cases, reported the inconsistent role of modular prosthesis exchange, and the nested study by Peel et al reported liner retention in approximately 40% of cases. We sought to overcome this by applying multiple imputation; however, ideally, more data are needed. Future studies should seek to apply standardized regiments, definitions, and surgical techniques and be sufficiently powered through multi-institutional collaboration to draw clearer conclusions.

The definition of PJI is controversial. Aligning with the CDC's classification for acute PJI,^25^ we included all infections that occurred within 12 months of the index TJA. However, Parvizi et al^38^ suggested 3 months as the cutoff for acute PJI, and other organizations, such as MSIS,^18^ ICM,^8^ and IDSA^19^ have issued alternative diagnostic criteria, which differentiate PJI as early onset (<3 months postsurgery), delayed onset (3 to 24 months postsurgery), and late onset (>24 months postsurgery). Because of this controversy, we conducted a sensitivity analysis using 3 months and 6 months as the cutoff time point for PJI, which yielded largely similar results to our primary analyses.

## Implications of Findings and Future Directions

Our findings suggested that male sex is a significant predictor of DAIR failure. Although this study only focused on sex, social factors, such as gender, are equally important and warrant further research. Another important implication of the study has been the development of an international collaboration, Orthopaedic Device Infection Network, involving academics, orthopaedic surgeons, and infectious disease specialists from Australia, New Zealand, Europe, and the United States. This enabled the linkage of multiple registry data to maximize epidemiological research. Currently, in Australia, there are no publicly available policies on evaluating and reporting sex-specific data, exacerbating the issue of a lack of consideration or awareness of sex in research.^39^ Updating policies, such as in research funding and publication requirements, is key for tackling the current issue. Finally, although the study analyzed outcomes after DAIR, further studies investigating sex differences for other treatment modalities, such as two-stage revision or amputation, would be useful to gain more insight into the complex interaction between sex and treating PJI.

## Conclusion

This is the first sex-specific IPD meta-analysis to assess the outcomes of DAIR in patients with hip and knee PJI. The current study can be used to raise awareness of the importance of sex differences in research and international collaborations to improve decision-making, patient counseling, and equitable health care.
